# Astrocyte-Targeted Transporter-Utilizing Derivatives of Ferulic Acid Can Have Multifunctional Effects Ameliorating Inflammation and Oxidative Stress in the Brain

**DOI:** 10.1155/2019/3528148

**Published:** 2019-11-13

**Authors:** Ahmed Montaser, Johanna Huttunen, Sherihan Abdelhamid Ibrahim, Kristiina M. Huttunen

**Affiliations:** ^1^School of Pharmacy, Faculty of Health Sciences, University of Eastern Finland, P.O. Box 1627, FI-70211 Kuopio, Finland; ^2^Department of Pharmacology and Therapeutics, Faculty of Pharmacy and Drug Manufacturing, Pharos University, 21311 Alexandria, Egypt

## Abstract

Ferulic acid (FA) is a natural phenolic antioxidant, which can exert also several other beneficial effects to combat neuroinflammation and neurodegenerative diseases, such as Alzheimer's disease. One of these properties is the inhibition of several enzymes and factors, such as *β*-site amyloid precursor protein (APP) cleaving enzyme 1 (BACE1), cyclooxygenases (COXs), lipoxygenases (LOXs), mammalian (or mechanistic) target for rapamycin (mTOR), and transcription factor NF-*κ*B. We have previously synthesized three L-type amino acid transporter 1- (LAT1-) utilizing FA-derivatives with the aim to develop brain-targeted prodrugs of FA. In the present study, the cellular uptake and bioavailability of these FA-derivatives were evaluated in mouse primary astrocytic cell cultures together with their inhibitory effects towards BACE1, COX/LOX, mTOR, NF-*κ*B, acetylcholinesterase (AChE), and oxidative stress. According to the results, all three FA-derivatives were taken up 200–600 times more effectively at 10 *μ*M concentration into the astrocytes than FA, with one derivative having a high intracellular bioavailability (*K*_p,uu_), particularly at low concentrations. Moreover, all of the derivatives were able to inhibit BACE1, COX/LOX, AChE, and oxidative stress measured as decreased cellular lipid peroxidation. Furthermore, one of the derivatives modified the total mTOR amount. Therefore, these derivatives have the potential to act as multifunctional compounds preventing *β*-amyloid accumulation as well as combating inflammation and reducing oxidative stress in the brain. Thus, this study shows that converting a parent drug into a transporter-utilizing derivative not only may increase its brain and cellular uptake, and bioavailability but can also broaden the spectrum of pharmacological effects elicited by the derivative.

## 1. Introduction

Most brain disorders and diseases lack effective drug therapies [1–3]. Current treatments of cerebral viral and bacterial infections as well as drugs against neurodegenerative and autoimmune diseases, such as Parkinson's disease (PD), Alzheimer's disease (AD), Huntington's disease (HD), amyotrophic lateral sclerosis (ALS), multiple sclerosis (MS), epilepsy, stroke, and traumatic brain injury, are largely unsatisfactory, often providing only symptomatic relief. One reason for the poor therapeutic responses in many central nervous system (CNS) diseases and disorders is due to ineffective drug delivery, i.e., the inability of a drug molecule to reach its target site at effective concentrations within the brain [4, 5]. The blood-brain barrier (BBB) prevents many drugs and other xenobiotics from gaining access to the brain, and therefore, several novel drug delivery technologies have been devised to overcome this obstacle. However, despite major industrial and academic efforts to develop novel drug delivery systems, this puzzle has remained largely unresolved. Furthermore, it was proposed over a decade ago that the cellular membranes of parenchyma cells could serve as a secondary barrier to drug permeability within the brain [6, 7]. However, this aspect has been neglected, even though a significant number of the novel CNS drugs have intracellular targets [8].

Inadequate understanding of the disease's etiology in the brain and poor translation from the animal models to the human situation have also contributed to the decelerated CNS drug development success rate [4]. Recently, oxidative stress and neuroinflammation have been proposed as potential mechanisms involved in the pathogenesis of several brain disorders and as possible targets in neuroprotective treatments [9, 10]. Since oxidative stress (i.e., overproduction of reactive oxygen species (ROS) and reactive nitrogen species (RNS)) as well as inflammation (i.e., overproduction of proinflammatory cytokines, such as TNF-*α*, IL-1*β*, and IL-6 as well as the overexpression of iNOS) can directly cause neuronal cell death or trigger a cascade of events that leads to further neurodegeneration (protein misfolding, mitochondrial dysfunction, or glial cell activation), the prevention of increased levels of ROS, RNS, and proinflammatory cytokines can not only treat the symptoms but also affect the progression of the neurodegenerative diseases. Natural polyphenols, which are compounds mainly found in plants, are known to be strong antioxidants and therefore can help prevent intracellular accumulation of ROS. Numerous studies have shown that they can exert multiple neuroprotective effects in neurological disorders and ischemia [11–13]. However, most of these polyphenols, like almost all small-molecular weight drugs, are unable to cross the BBB and reach their intracellular target sites within the brain [14].

**Figure 1:**
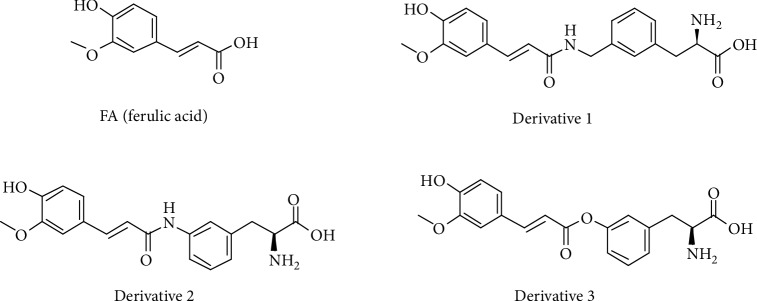
Ferulic acid (FA) and its L-type amino acid transporter 1- (LAT1-) utilizing derivatives **1**-**3**.

One natural phytochemical, ferulic acid (FA) (*trans-*4-hydroxy-3-methoxycinnamic acid; Figure 1), found in fruits and vegetables, is a phenolic antioxidant [15]. In addition to being a free radical scavenger against the ROS produced during inflammation within the brain, it has also been reported to ameliorate memory deficits in the amyloid-*β*- (A*β*_1-42_-) induced Alzheimer's disease (AD) mouse model by inhibiting *β*-site amyloid precursor protein (APP) cleaving enzyme 1 (BACE1) activity [16]. This was found to prevent further A*β*_1-42_ accumulation and to help to lessen the subsequent activation of astrocytes and microglial cells [17, 18]. We have previously reported that L-type amino acid transporter- (LAT1-) utilizing prodrugs of FA can display enhanced delivery across the BBB and thus can be accumulated with the brain [19]. However, a previous study also revealed that the prodrugs that were conjugated to FA via an amide bond (derivatives **1** and **2** in Figure 1) were not capable of releasing FA at a sufficient rate within the mouse brain *in vivo*. FA-derivative **1** did not release any FA, while FA-derivative **2** released less than 5% of FA in the brain. The corresponding ester derivative of FA (derivative **3** in Figure 1) had a more favorable bioconversion rate *in vitro*; in mouse brain S9 subcellular fraction, it released approximately 30% of FA during a 5 h incubation, while derivatives **1** and **3** remained intact. However, due to the very high carboxylesterase activity in the plasma and in the hepatocytes, the ester prodrug was not able to deliver FA into the mouse brain; instead, it was prematurely bioconverted to FA by the first pass metabolism. In contrast, in human plasma and liver S9 subcellular fraction, prodrug **3** was 10 times more stable. Due to their higher stability, previously called prodrugs **1**-**3** will be considered here as FA-derivatives. Thus, the aim of the present study was to evaluate the detailed transport mechanisms of the LAT1-utilizing derivatives of FA into their target brain cells, namely, mouse primary astrocytes [20], and investigate their effects on inflammation and oxidative stress. This is highly important information in determining whether these compounds could be used as therapeutic agents in their intact form and not as prodrugs.

## 2. Experiments

### 2.1. Materials

All reagents and solvents used in analytical studies were commercial and high purity of analytical grade or ultragradient HPLC-grade purchased from Sigma (St. Louis, MO, USA), J.T. Baker (Deventer, The Netherlands), Merck (Darmstadt, Germany), Riedel-de Haën (Seelze, Germany), or Thermo Fisher Scientific, Inc. (Waltham, MA, USA). Water was purified using a Milli-Q Gradient system (Millipore, Milford, MA, USA).

### 2.2. Cell Cultures

Primary astrocytes from the cortex and hippocampi were isolated from 2-day-old wild-type and *APP/PS1* transgenic mice as previously described [21, 22]. Mice carrying human *APP* (K595N and M596L) and *PSEN1dE9* mutations maintained in C57BL/6J background were used as a mouse model of AD (Jackson Laboratories, Bar Harbor, ME, USA). The animals were housed and treated as described above, and cortices and hippocampi were isolated by suspending the brain tissue into DMEM medium containing 10% heat-inactivated fetal bovine serum and penicillin streptomycin (100 U/mL). The suspension was triturated ten times and thereafter centrifuged at 1500 rpm for 5 min at room temperature. Trypsin-EDTA of 0.25% was added, and the suspension was incubated for 30 min at 37°C. Fresh culture medium was added, and the suspension was centrifuged at 1500 rpm for 5 min. The astrocytes were cultured in Dulbecco's modified Eagle medium/F-12 Nutrient Mixture (DMEM/F2) supplemented with L-glutamine (2 mM), heat-inactivated fetal bovine serum (10%), penicillin (50 U/mL), and streptomycin (50 *μ*g/mL). The cells were plated on a poly-D-lysine coated flasks in culture medium, and to remove the microglia, the cultures were shaken at 200 rpm for 2 h before the experiments described below were performed. It has been reported earlier that these cultures contain approximately 80% astrocytes (20% microglia) [23].

### 2.3. Ability of Compounds to Bind to LAT1

For the transporter binding studies, the astrocytes were seeded on 24-well plates with a density of 10 × 10^4^ cells/well three days before the experiments. After removal of the culture medium, the cells were carefully washed with prewarmed HBSS (Hank's balance salt solution) containing choline chloride (125 mM), KCl (4.8 mM), MgSO_4_ (1.2 mM), KH_2_PO_4_ (1.2 mM), CaCl_2_ (1.3 mM), glucose (5.6 mM), and HEPES (25 mM) adjusted to pH 7.4 with 1 M NaOH. The cells were preincubated with 500 *μ*L of prewarmed HBSS at 37°C for 10 min before the experiments. To study the ability of studied compounds to inhibit the uptake of a known LAT1 substrate, the cells were incubated at 37°C for 5 min with an uptake buffer (HBSS, 250 *μ*L) containing 0.76 *μ*M (0.25 mCi/mL or 9.85 MBq/mL) of [^14^C]-L-leucine (PerkinElmer, Inc., Waltham, MA, USA) and 0.1-1000 *μ*M of studied compound (or HBSS as blank). After incubation, the uptake was stopped by adding 500 *μ*L of ice-cold HBSS and the cells were washed two times with ice-cold HBSS. The cells were then lysed with 500 *μ*L of NaOH (0.1 M) for 60 min, and the lysate was mixed with 3.5 mL of Emulsifier safe cocktail (Ultima Gold, PerkinElmer, Inc., Waltham, MA, USA). The radioactivity in the cells was measured by liquid scintillation counting (MicroBeta^2^ counter, PerkinElmer, Inc., Waltham, MA, USA). Half of maximum inhibitory concentration (IC_50_) values were calculated by nonlinear regression analysis (fitting the curve to log (concentration) vs. remaining normalized viability).

### 2.4. Transporter-Mediated Cellular Uptake of Compounds

For the cell uptake experiments, the astrocytes were seeded on 24-well plates with a density of 10 × 10^4^ cells/well three days before the experiments as described earlier [24]. Cellular uptake of derivatives **1**-**3** of FA was studied by incubating the cells at 37°C for 30 min (uptake was linear with all compounds up to 30 min) with compounds at the concentration of 1-200 *μ*M in prewarmed HBSS buffer (250 *μ*L). Subsequently, the cells were washed three times with ice-cold HBSS and lysed with 250 *μ*L of NaOH (0.1 M) for 60 min. The lysates were diluted with acetonitrile (ACN) including the selected internal standard (diclofenac to compounds **1**-**3** and chlorzoxazone to FA) with a ratio of 1 : 3 and centrifuged at 10 000 × *g* for 10 min. The samples were analyzed by the liquid chromatography mass spectrometry (LC-MS) methods described earlier for derivatives **1-3** and FA with an Agilent 1200 Series Rapid Resolution LC System together with an Agilent 6410 Triple Quadrupole Mass Spectrometer equipped with an electrospray ionization source using a Poroshell 120 EC-C-18 column (50 mm × 2.1 mm, 2.7 *μ*m; Agilent Technologies, Santa Clara, CA) [19]. The concentrations of each compound in cell lysates were calculated from the standard curve that was prepared by spiking known amounts of compounds to ACN including the selected internal standard and then normalized with protein concentration. The protein concentrations on each plate were determined as a mean of three samples by Bio-Rad Protein Assay, based on the Bradford dye-binding method, using bovine serum albumin (BSA) as a standard protein and measuring the absorbance (595 nm) by a multiplate reader (EnVision, PerkinElmer, Inc., Waltham, MA, USA).

### 2.5. Intracellular Unbound Concentrations of Compounds

The unbound fraction of FA and its derivatives **1**-**2** was determined at 10 *μ*M in cell homogenate by using a Rapid Equilibrium Dialysis (RED) device (Thermo Fisher Scientific, Inc., Waltham, MA, USA). Briefly, the cell homogenate was prepared from astrocyte cell suspension (10 × 10^6^ cells/mL) in HBSS with a SoniPerp 150 Plus disintegrator (MSE Ltd., London, UK) for 2 s × 3. 100 *μ*L of cell homogenate spiked with the studied compound was added to the reaction chamber, and 350 *μ*L of HBSS buffer was added to the buffer chamber of the RED plate. The dialysis plate was incubated at 37°C for 4 h with shaking. 50 *μ*L of samples was taken from the reaction, and buffer chambers and equal size of buffer or blank homogenate were added, respectively, to yield identical matrices. The proteins were precipitated by adding 100 *μ*L of ice-cold ACN (including the selected internal standard; see above), and the supernatants were collected for LC-MS analysis (see above) after centrifugation at 12 000 × *g* for 10 min. The unbound drug fraction (*f*_u,cell_) was calculated as described by Mateus et al. [25]:

(1) $$\begin{array}{c} f_{\text{u},\text{homogenate}}=\frac{C_{\text{buffer}}}{C_{\text{homogenate}}}, \end{array}$$

where *C*_buffer_ is compound concentration in the buffer chamber and *C*_homogenate_ is the compound concentration in the homogenate chamber and corrected with homogenate dilution factor (*D*) [26]:

(2) $$\begin{array}{c} f_{\text{u},\text{cell}}=\frac{1}{D\times \left(1/f_{\text{u},\text{homogenate}}-1\right)+1}, \end{array}$$

where *D* was estimated to be 45 for 10 × 10^6^ cells/mL cell suspension according to their weight of the cells.

The drug concentration ratio in astrocytes (*K*_p_) was determined at 0.01-1.0 *μ*M concentrations by comparing the cellular uptake from the cell lysates (0.1 M NaOH) to the concentration detected from the surrounding buffer (HBSS) collected before cell lysing as described by Mateus et al. [25]:

(3) $$\begin{array}{c} K_{\text{p}}=\frac{A_{\text{cell}}/V_{\text{cell}}}{C_{\text{medium}}}, \end{array}$$

where *A*_cell_ is the amount of compound in the cell lysate (nmol), *V*_cell_ is the astrocyte cell volume (0.05810^−15^ L/cell) [27] for 10 × 10^4^ cells/well, and *C*_medium_ is the compound concentration in the surrounding buffer. The unbound drug concentration ratio (*K*_p,uu_) was then calculated by multiplying the drug concentration ratio (*K*_p_) by unbound drug fraction (*f*_u,cell_) as described by Mateus et al. [25]:

(4) $$\begin{array}{c} K_{\text{p},\text{uu}}=K_{\text{p}}\times f_{\text{u},\text{cell}}. \end{array}$$

### 2.6. Ability of Compounds to Inhibit Astrocyte Cell Growth

Primary astrocytes were seeded at the density of 10 × 10^3^ cells/well onto collagen-coated 96-well plates. The cells were used for the proliferation experiments one day after seeding. Concentrations of 5-400 *μ*M FA or its LAT1-utilizing derivatives **1**-**3** were added into the growth medium and incubated for 3 days. Each day, the cell viability was determined by resazurin cell proliferation kit (Sigma, St. Louis, MO, USA), which is directly proportional to aerobic respiration and cellular metabolism of cells. The samples were measured fluorometrically by monitoring the increase in fluorescence at *λ*_ex_ 560 nm and *λ*_em_ 590 nm (EnVision, PerkinElmer, Inc., Waltham, MA, USA). The cell viability was also followed by visualizing the wells with microscopy. The ability of compounds to inhibit the viability of the cells was expressed as percentages (%) compared to the untreated controls.

### 2.7. Ability of Compounds to Inhibit Oxidative Stress and Lipid Peroxidation (Malondialdehyde Formation)

Primary astrocytes were seeded at the density of 2 × 10^5^ cells/well onto collagen-coated 6-well plates. The cells were used for the experiments 2 days after seeding, and first, they were treated with 0.1 *μ*g/mL lipopolysaccharide (LPS) in prewarmed HBSS buffer (250 *μ*L) at 37°C for 24 h. The control cells were treated with prewarmed HBSS buffer only. The ability of compound to inhibit the oxidative stress was evaluated by adding FA (50 *μ*M) or its derivatives **1**-**3** in prewarmed HBSS buffer (250 *μ*L) together with LPS and incubating the cells at 37°C for 24 h. Tocopherol (vitamin E) was used as a positive control. Subsequently, the cells were washed with PBS (phosphate-buffered saline), detached from the plate by trypsinization and centrifuged at 1000 × *g* for 5 min. The supernatant was removed, and the cell pellet was then resuspended with 0.1 M MES (2-(N-morpholino)ethanesulfonic acid) buffer (pH 6.0), sonicated for 10 min, and centrifuged at 10 000 × *g* for 15 min at 4°C. The supernatant (100 *μ*L) was collected, and 100 *μ*L glacial acetic acid (50%), 4 *μ*L of butylated hydroxytoluene (0.01%), and finally 100 *μ*L of thiobarbituric acid (4 mM) were added and the mixture was incubated in a thermoshaker at 95°C for 1 h. The mixtures were then centrifuged at 10 000 × *g* for 10 min, and 50 *μ*L of the supernatant was analyzed on a 96-well plate, each experiment as three technical replicates from three biological replicates. The fluorescence was read by the EnVision plate reader (EnVision, PerkinElmer, Inc., Waltham, MA, USA) at *λ*_ex_ 532 nm and *λ*_em_ 553 nm. The amount of produced malondialdehyde (MDA) in each experiment was quantified from the standard curve; various amounts (1-800 *μ*M) of MDA and thiobarbituric acid in water were incubated in a thermoshaker at 95°C for 1 h and analyzed simultaneously as duplicates with each studied batch. The protein concentrations on each plate were determined as a mean of three samples by Bio-Rad Protein Assay, based on the Bradford dye-binding method, using BSA as a standard protein and measuring the absorbance (595 nm) by a multiplate reader (EnVision, PerkinElmer, Inc., Waltham, MA, USA). The results were analyzed as *μ*mol of formed MDA per mg protein.

### 2.8. Ability of Compounds to Inhibit BACE1

A fluorometric assay was used to screen the inhibitory effect of the studied compounds on purified human BACE1 using a fluorescence resonance energy transfer (FRET) peptide technique (SensoLyte© 520 BACE1 Assay kit, AnaSpec, Inc., Fremont, CA, USA) according to the manufacturer's protocol. Briefly, 1 *μ*M and 10 *μ*M of studied compounds were incubated with the FRET substrate (QXL® 520/HiLyte™ Fluor 488) and active BACE1 enzyme at room temperature for 30 minutes. The sequence of the FRET peptide has been derived from the *β*-secretase cleavage site of *β*-amyloid precursor protein (APP) with Swedish mutation, which enhances *β*-secretase to process APP resulting in an early onset of AD. Thus, active *β*-secretase cleaved FRET substrate into two separate fragments resulting in the release of HiLyte™ Fluor 488 fluorescence that was measured by the EnVision plate reader (EnVision, PerkinElmer, Inc., Waltham, MA, USA) at *λ*_ex_ 490 nm and *λ*_em_ 520 nm. Changes in the amount of this fluorophore caused by the inhibition of *β*-secretase by the studied compounds (1-10 *μ*M) were compared with the control sample and with a specific inhibitor (0.25 *μ*M provided with the kit) (KTEEISEVN-Sta-VAEF-NH_2_) [28]. The results were reported as percentages (%), with control sample representing 100% *β*-secretase activity.

### 2.9. Ability of Compounds to Inhibit Peroxidase Activity of Cyclooxygenases

A coupled oxidation-reduction fluorometric assay was used to assess the inhibitory effect of compounds (100 *μ*M) together with positive controls on the peroxidase activity of cyclooxygenases (COX) (ab204699 Cyclooxygenase Activity Assay Kit, Abcam, Cambridge, UK). The method has been done and validated according to the manufacturer's protocol. Briefly, studied compounds (100 *μ*M) and the reaction mixture were incubated with the cell lysates at room temperature and the reaction was initiated by adding arachidonic acid/NaOH solution. The fluorescence was read in a kinetic mode after every 15 seconds for 30 minutes by the EnVision plate reader (EnVision, PerkinElmer, Inc., Waltham, MA, USA) at *λ*_ex_ 535 nm and *λ*_em_ 587 nm.

### 2.10. Ability of Compound to Inhibit the Activity of Acetylcholinesterase and Butyrylcholinesterase

Inhibitory activities of FA and its derivatives **1**-**3** towards acetylcholinesterase (AChE) and butyrylcholinesterase (BChE) were determined with an endpoint enzymatic assay in mouse brain S9 fraction by Ellman's method by using acetylthiocholine (1 mM) for measuring AChE activity and butyrylthiocholine (1 mM) for measuring BChE activity. Briefly, mouse brain S9 fraction was prepared by homogenizing freshly collected mouse brain with 50 mM Tris-buffered saline (TBS) (pH 7.4) (1 : 4, *w*/*v*), centrifuging the homogenate at 9 000 × *g* for 20 min at 4°C and collecting the supernatant. The supernatant was then diluted at 1 : 10 with phosphate-buffered saline (100 mM; pH 7.0) and mixed with Ellman's reagent (5,5′-dithiobis-(2-nitrobenzoic acid); DTNB; 1 mM) and studied compounds in DMSO (DMSO concentration was less than 0.5%) on a 96-well plate as 3 parallel assays. After reading the absorbance by the EnVision plate reader (EnVision, PerkinElmer, Inc., Waltham, MA, USA) at 412 nm, acetylthiocholine or butyrylthiocholine was added and shaken and the enzymatic activities of AChE or BChE were read at the intervals of 5 min until 30 min. The concentration of studied compounds required to inhibit the specific activity of AChE or BChE (*μ*mol/min/mg protein) was evaluated at a concentration range of 1-800 *μ*M and presented as a half of maximum inhibitory concentration (IC_50_) for each compound in each biological media at the end point (30 min). Unselective ChE inhibitor tacrine and AChE-selective inhibitor donepezil were used as a positive controls in both assays.

The detailed types of inhibition of FA-derivatives **1**-**3** were evaluated by using 20-1000 *μ*M concentrations of AChE substrate, acetylthiocholine, in the presence of 16, 25, or 66 *μ*M of FA-derivatives **1**-**3**, respectively (according to their IC_50_ values; Section 3.8). According to the Hanes-Woolf plots, the ratio of initial substrate concentration to the reaction velocity ([*S*]/*v*) was plotted against the substrate concentration (*μ*M). The linear regression was used to calculate the *K*_m_ value (negative value of x-intercept) and *V*_max_ value (1/slope).

### 2.11. Ability of Compounds to Affect Total Amounts of mTOR and NF-*κ*B

Enzyme-Linked Immunosorbent Assay (ELISA) kits were used (mTOR SimpleStep ELISA Kit and *NFκB* p65 Total SimpeStep ELISA Kit, Abcam, Cambridge, UK) to quantify mammalian (or mechanistic) target of rapamycin (mTOR) and transcription factor NF-*κ*B amounts, respectively. The compounds (100 *μ*M) were incubated for 48-96 hours with the cells at a density of 2 × 10^5^ in 6-well plates. The control wells were treated with the solvent only (0.5% DMSO). Cells were solubilized using the provided extraction buffer with the kit, incubated on ice for 20 minutes and centrifuged at 18 000 × *g* for 20 min at 4°C. The supernatants were stored at -80°C till the day of the analysis. Standards and samples were then analyzed following the manufacturer's protocol (ELISA sandwich method) and by reading the absorbance with the EnVision plate reader (EnVision, PerkinElmer, Inc., Waltham, MA, USA) at 450 nm. The results were analyzed as pmol of formed mTOR or NF-*κ*B per mL.

### 2.12. Data Analysis

All the cellular studies were carried out as three biological replicates from the same cell passage. The function of LAT1 was followed between the used cell passages (7-16) with a LAT1 probe substrate, [^14^C]-L-leucine, and noticed to be unaltered. All statistical analyses, including Michaelis-Menten, Eadie-Hofstee, and Hanes-Woolf analyses, were performed using GraphPad Prism v. 5.03 software (GraphPad Software, San Diego, CA, USA). Statistical differences between groups were tested using one-way ANOVA, followed by Tukey's multiple comparison test, and presented as the mean ± SD, with significant differences denoted by asterisks (^∗^*P* < 0.05, ^∗∗^*P* < 0.01, and ^∗∗∗^*P* < 0.001).

### 2.13. Ethical Statement

The experimental procedures involving animals (primary neuron and astrocyte isolation) were made in compliance with the European Commission Directives 2010/63/EU and 86/609 and approved by the Institutional Animal Care and Use Committee of University of Eastern Finland (Animal Usage Plan numbers EKS-008-2016, EKS-006-2017, and ESAVI/3347/04.10.07/2015). All efforts were made to minimize the number of animals used and to minimize their suffering.

## 3. Results

### 3.1. Ability of LAT1-Utilizing Derivatives of Ferulic Acid to Bind LAT1 in Astrocytes

The ability of FA and its derivatives **1**-**3** to bind to LAT1 was studied in mouse primary astrocytes, from which we have recently characterized LAT1 expression and function [29]. All three derivatives of FA (**1**-**3**) were able to bind to LAT1 and inhibit the uptake of a LAT1-probe substrate, [^14^C]-L-leucine, at low to very low (micromolar) concentrations (Table 1). The amide derivative **1** had the greatest affinity for LAT1, and its IC_50_ value was lower (2.2 *μ*M) in astrocytes as compared to previously determined values in human breast adenocarcinoma cells (MCF-7; 14.2 *μ*M), while the opposite was observed for derivative **2** (8.9 *μ*M vs. 1.1 *μ*M in MCF-7). The ester analogue **3** had higher IC_50_ values than amide derivatives **1**-**2**. This was most likely due to the premature bioconversion of the ester derivative to the parent drug on the cell surface, since the parent drug, FA, was not able to bind to LAT1.

### 3.2. Uptake of LAT1-Utilizing Derivatives of Ferulic Acid into Astrocytes via LAT1

Cellular uptake of FA and its amide derivatives **1** and **2** and ester derivative **3** into primary astrocytes was concentration-dependent ([Supplementary-material supplementary-material-1]). The uptake of FA-derivatives was 2–10 times higher than that of FA at 100 *μ*M and 210–660 times higher at 10 *μ*M concentrations (Table 2). The Eadie-Hofstee analysis revealed, however, that derivatives **1** and **2** were utilizing two separate transporters, the high affinity-low capacity transporter, LAT1, and another lower affinity-higher capacity transporter ([Supplementary-material supplementary-material-1]). Curiously, FA and derivative **3** had an autoactivated Eadie-Hofstee profile. This means that FA and derivative **3** are able to induce LAT1 function or to increase its expression on the plasma membrane [30]. The affinity for LAT1-mediated uptake of FA-derivatives **1**-**2** was high (*K*_m_ values approximately 4 *μ*M), while the affinity for the secondary transport mechanism was much lower (*K*_m_ values of 100-150 *μ*M) (Table 2). Derivatives **1**-**2** were also more effectively transported into the astrocytes via the secondary transport mechanism (30–170 pmol/min/mg protein) than via LAT1 (8-13 pmol/min/mg protein). We have previously reported that this secondary transport mechanism is most likely the organic anion transporting polypeptides (OATPs) or the organic anion transporters (OATs), since a similar uptake pattern has also been observed in human breast cancer cells (MCF-7) and this uptake is known to be probenecid-sensitive [19, 31]. However, due to the 30–40 times higher affinity for LAT1, the role of this secondary transport mechanism in the total cellular uptake of these compounds at clinically relevant concentration (i.e., <100 *μ*M) was considered to be minor, perhaps even nonexistent.

### 3.3. Intracellular Unbound Drug Concentrations of LAT1-Utilizing Derivatives of Ferulic Acid

**Figure 2:**
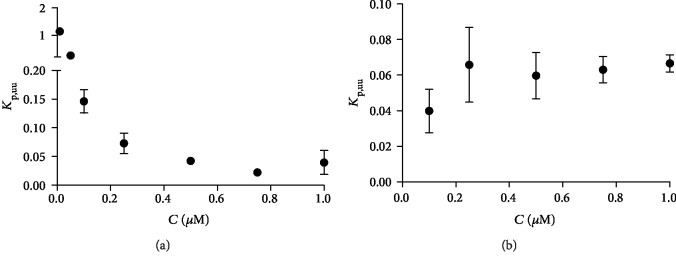
Intracellular unbound fractions (*K*_p,uu_ values) of (a) FA-derivative **1** and (b) FA-derivative **2** plotted in the studied concentration range (0.01-1 *μ*M). Data are presented as the mean ± SD (*n* = 3).

The majority of the drug targets are localized intracellularly [8]. Therefore, it is the intracellular unbound drug concentrations that are considered to be pharmacologically relevant, and thus, it is important to evaluate drugs' ability to interact with their final target(s). In this study, we utilized the intracellular kinetic method that has been previously proposed by Mateus et al., which combines drug's intracellular binding and steady-state intracellular total drug concentration [25]. As a result, one can estimate drug's unbound intracellular : extracellular ratio, which can be reported as the *K*_p,uu_ value. In this study, FA-derivative **1** had higher *K*_p,uu_ values, particularly at lower concentrations (<0.1 *μ*M; 0.25-2.14) as compared to FA-derivative **2**, which in turn had relatively similar *K*_p,uu_ values throughout the studied concentrations (0.04-0.08 *μ*M; 0.83-1.74) (Figure 2). The unbound fraction of FA-derivative **1** was also slightly higher (*f*_u_ value of 16.26 ± 2.72) than the corresponding value of FA-derivative **2** (*f*_u_ value of 8.06 ± 0.54) ([Supplementary-material supplementary-material-1]). This clearly demonstrates that although the cellular total uptake of FA-derivative **2** was higher than that of FA-derivative **1** (Table 2, [Supplementary-material supplementary-material-1]), the comparison of the intracellular unbound concentrations proves that the FA-derivative **1** may be a more promising drug candidate for further studies. Due to its faster metabolism, *f*_u_ of derivative **3** was not evaluated, and therefore, the *K*_p,uu_ value for derivative **3** is not reported in the present study. Unfortunately, the cellular uptake of FA was also minor, and we were not able to quantify it at lower concentrations (0.1-1 *μ*M). Thus, it was not possible here to compare the *K*_p,uu_ value of FA against the FA-derivatives. However, due to its negligible cellular uptake, we can conclude that the intracellular unbound concentration of FA would also be extremely small, although there was no evidence that it was bound nonspecifically to other cell components, i.e., proteins and lipids (*f*_u_ value of 100%; [Supplementary-material supplementary-material-1]).

### 3.4. Ability of Ferulic Acid and Its LAT1-Utilizing Derivatives to Inhibit Astrocyte Cell Growth

To evaluate if FA and its derivatives **1**-**3** can affect the viability of the primary astrocytes, the compounds were incubated at 72 h with variable concentrations (5-400 *μ*M). FA and its amide derivatives **1** and **2** as well as the ester derivative **3** did not display any antiproliferative efficacy on the astrocytes at lower concentrations but did exhibit significant antiproliferative efficacy at concentrations above 200 *μ*M ([Supplementary-material supplementary-material-1]). Interestingly, FA and its derivatives **1** and **2** increased the viability of these cells at low concentrations (5-100 *μ*M). Therefore, these derivatives can be considered safe or at least nontoxic to astrocytes, when administered at clinically relevant concentrations.

### 3.5. Ability of Ferulic Acid and Its LAT1-Utilizing Derivatives to Inhibit Oxidative Stress and Lipid Peroxidation

**Figure 3:**
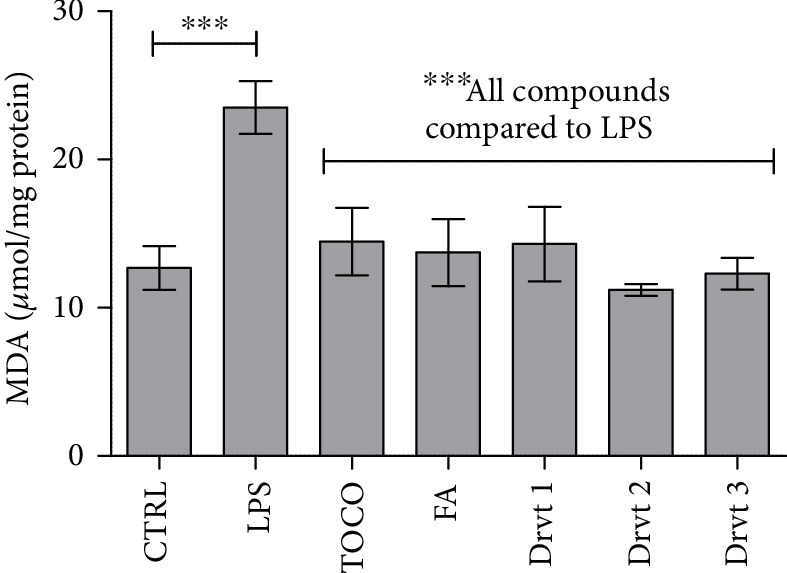
Inhibitory effects of 50 *μ*M ferulic acid (FA) and its LAT1-utilizing derivatives **1**-**3** as well as tocopherol (TOCO; vitamin E) on lipopolysaccharide- (LPS-) induced lipid peroxidation measured as malondialdehyde (MDA) formation after 24 h incubation. Data are presented as the mean ± SD (*n* = 3). An asterisk denotes a significant difference from the respective control (^∗∗∗^*P* < 0.001, one-way ANOVA, followed by Tukey's test).

The ability of FA and its derivatives **1**-**3** to inhibit oxidative stress and subsequent lipid peroxidation was evaluated with primary astrocytes by incubating the cells with 0.1 *μ*g/mL LPS for 24 h with and without the studied compounds and determining malondialdehyde formation (MDA) as previously described for FA [32]. As expected, 0.1 *μ*g/mL LPS increased the lipid peroxidation in astrocytes significantly from 12.69 ± 1.47 to 23.52 ± 1.77 *μ*mol/mg protein (185%) (Figure 3). Coincubation of LPS with the studied compounds: 50 *μ*M tocopherol (as positive control), FA, or its LAT1-utilizing derivatives **1**-**3,** inhibited the LPS-induced lipid peroxidation by 39–52%. Moreover, the lipid peroxidation measured in these cotreatments was comparable to the control level (11.21 ± 0.41–14.46 ± 2.28 *μ*mol/mg protein). Curiously, the MDA levels were approximately 12% lower after derivative **2** treatment than the MDA levels in the control incubation. Nevertheless, there was no significant difference in the inhibitory activity between derivatives **1**-**3**.

### 3.6. Ability of Ferulic Acid and Its LAT1-Utilizing Derivatives to Inhibit BACE1

**Figure 4:**
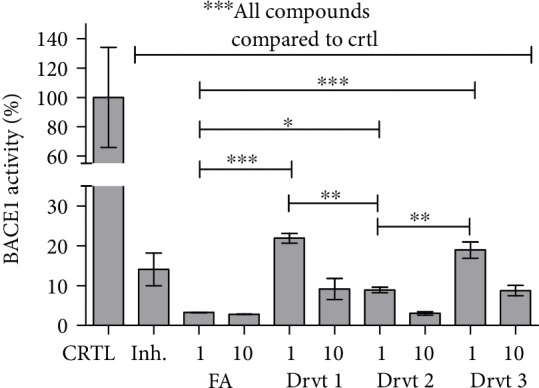
Inhibitory effects of 1 or 10 *μ*M ferulic acid (FA) and its LAT1-utilizing derivatives **1**-**3** and 0.25 *μ*M BACE1 inhibitor (KTEEISEVN-Sta-VAEF-NH_2_) [28] on inhibiting the *β*-site amyloid precursor protein (APP) cleaving enzyme 1 (BACE1) activity after 30 min incubation. Data are presented as the mean ± SD (*n* = 3). An asterisk denotes a significant difference from the respective control (^∗^*P* < 0.05, ^∗∗^*P* < 0.01, and ^∗∗∗^*P* < 0.001, one-way ANOVA, followed by Tukey's test).

Since FA itself has been reported to modulate directly BACE1 activity [16–18], it was also evaluated in the present study if the FA-derivatives would exert similar effects on BACE1. FA as well as all the derivatives **1**-**3** proved to be BACE1 inhibitors (Figure 4). The inhibitory efficacy of derivative **2** was comparable to FA at 1 and 10 *μ*M concentrations (ca. 91–97% vs. 97%, respectively), while the other derivatives (**1** and **3**) were significantly less potent than FA and derivative **2** (inhibition ca. 78–91 and 81–91%, respectively). Interestingly, 1 *μ*M FA and derivative **2** inhibited BACE1 more effectively than 0.25 *μ*M inhibitor (KTEEISEVN-Sta-VAEF-NH_2_) [28], provided by the kit (inhibition ca. 85%).

### 3.7. Ability of Ferulic Acid and Its LAT1-Utilizing Derivatives to Inhibit Peroxidase Activity

**Figure 5:**
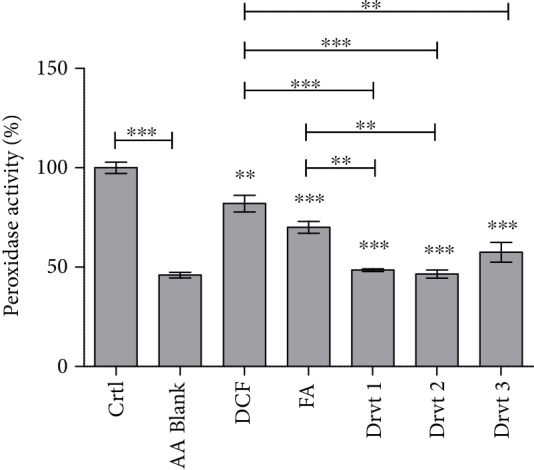
Inhibitory effects of 100 *μ*M ferulic acid (FA) and its LAT1-utilizing derivatives **1**-**3** as well as diclofenac (DCF) on peroxidase activity of cyclooxygenases (COX) after 30 min incubation. Data are presented as the mean ± SD (*n* = 3). An asterisk denotes a significant difference from the respective control (^∗∗^*P* < 0.01 and ^∗∗∗^*P* < 0.001, one-way ANOVA, followed by Tukey's test).

It has also been reported that FA can revert the hypoxia-induced COX-2 expression and inhibit the production of prostaglandin E2 (PGE2) within a concentration-dependent manner [33]. Therefore, the ability of FA-derivatives **1**-**3** to inhibit also COX enzymes was evaluated in the present study. Both FA and derivatives **1**-**3** inhibited the cellular peroxidase activity of COX enzymes (Figure 5). It is noteworthy that the inhibition by FA-derivatives was significantly higher (43-54%) than with FA itself or a known COX inhibitor, diclofenac (DCF). The inhibition by DCF was only 18% in this assay, and thus, it was almost 3 times lower than with the novel FA-derivatives. However, one needs to keep in mind that the assay we used in the present study is not able to differentiate the peroxidase activity of COX enzymes from the lipoxygenases (LOX) and it has been reported that ferulic acid and its derivatives can also inhibit at least 5- and 15-LOX [34, 35]. Therefore, since FA-derivatives **1**-**3** had a higher ability to inhibit peroxidase activity than a clinically used COX inhibitor DCF, it is highly likely that these derivatives can inhibit both the production of prostaglandins and that of the leukotrienes, which broadens their multifunctional properties even wider. Nevertheless, no significant difference among FA-derivatives **1**-**3** was observed in this assay.

### 3.8. Ability of Ferulic Acid and Its LAT1-Utilizing Derivatives to Inhibit AChE/BuChE Activity

We have reported earlier that a LAT1-utilizing prodrug, ketoprofen, was a substrate of AChE, as measured by its IC_50_ value against AChE activity in rat brain S9 fraction [36]. Furthermore, this bioconversion in rat brain S9 fraction was inhibited by donepezil, a selective inhibitor of AChE. Therefore, we wanted to evaluate if the derivatives of FA could also interact with AChE. All of the studied FA-derivatives (**1**-**3**) inhibited the activity of AChE in mouse brain S9 subcellular fraction, whereas FA itself did not have any effect (Table 3). Furthermore, amide derivatives **1** and **2** were able to inhibit AChE at micromolar concentrations, although the inhibitions were not as significant as with donepezil (IC_50_ values of 16-25 *μ*M compared to 17 nM, respectively). None of the studied compounds inhibited the activity of BuChE, although FA-derivatives **1** and **3** were able to bind to the substrate binding site of brain BuChE with very low affinity (over 220 *μ*M IC_50_ value; Table 3). The linear regression of Hanes-Woolf plots showed that all of the derivatives inhibited AChE in a mixed type manner, as the affinity for acetylthiocholine was increased: *K*_m_ from 130 to 187-324 *μ*M, respectively, while the velocity of the reaction was decreased from 2.21 nmol/min/mg protein to 2.12-2.09 nmol/min/mg protein, respectively ([Supplementary-material supplementary-material-1] with derivatives **1** and **3**). In addition to being able to bind to AChE, the bioconversion of derivative **3** in mouse brain S9 subcellular fraction was slowed down in the presence of donepezil, from a half-life of approximately 215 min to a level in which no bioconversion at all was observed. This indicates that AChE is able to slowly convert FA-derivative **3** into FA in mouse brain.

### 3.9. Ability of Ferulic Acid and Its LAT1-Utilizing Derivatives to Affect Total Amounts of mTOR and NF-*κ*B

**Figure 6:**
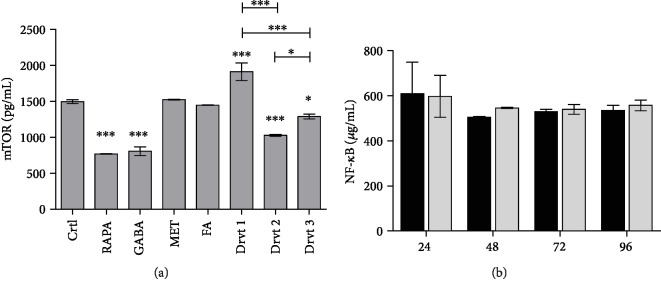
(a) Inhibitory effects of 100 *μ*M ferulic acid (FA) and its LAT1-utilizing derivatives **1**-**3** as well as those of 1 *μ*M rapamycin or 100 *μ*M gabapentin or metformin on mammalian target of rapamycin (mTOR) total amount after 48 h incubation. (b) The effects of 100 *μ*M FA-derivative **2** on the total amount of NF-*κ*B after incubation for 24, 48, 72, and 96 h. Data are presented as the mean ± SD (*n* = 3). An asterisk denotes a significant difference from the respective control (^∗^*P* < 0.05 and ^∗∗∗^*P* < 0.001, one-way ANOVA, followed by Tukey's test).

mTOR is a key regulator of the autophagy-lysosomal pathway, and thus, it has a critical role in regulating intra- and extracellular levels of A*β* in brain parenchyma [37]. Moreover, it has been reported that increased amounts of mTOR are related to elevated levels of A*β* in AD mouse brain. In addition, a natural polyphenol, resveratrol, which is structurally related to FA and an inhibitor of mTOR, has been reported to have beneficial effects against autophagy-lysosomal degradation of A*β*. It is also known that in addition to its antioxidant properties, 1 mM FA can also exert inhibitory activity on cellular mTOR signaling pathways [38]. Therefore, the inhibitory activities of FA-derivatives **1**-**3** (100 *μ*M) were evaluated in this study. FA-derivatives **2** and **3** reduced the total amount of mTOR after 48 h coincubation with the cells, along with 1 *μ*M rapamycin or 100 *μ*M gabapentin or metformin as positive controls (Figure 6(a)). Derivative **2** had a comparable effect (approximately 32%) to rapamycin and gabapentin (46-49%), and thus, it was significantly more potent than the other FA-derivatives. Derivative **3** reduced the total amount mTOR protein only moderately (approximately 14%), while ferulic acid itself and another positive control, metformin, did not affect to the total amount of mTOR. Moreover, FA-derivative **1** increased the amount of mTOR (approximately 28%). These discrepancies may stem from the fact that in this study we were determining the total protein amount of mTOR and not its activity, which could be measured via downstream signaling biomarkers. Thus, in the future, it would be important to clarify the exact mechanism of FA-derivatives on mTOR signaling pathways.

Transcription factor NF-*κ*B is recognized as an important agent related to inflammation in the AD brain, since A*β*_1-42_ has been shown to activate NF-*κ*B in astrocytes, which in turn has led to the increased production of proinflammatory cytokines, such as TNF-*α*, IL-1*β*, and IL-6, as well as overexpression of iNOS [39]. Since mTOR signaling can also affect NF-*κ*B via the Akt serine-threonine kinase pathway [40], it was hypothesized that FA-derivative **2**, which had the greatest effect on the amount of mTOR protein (Figure 6(a)), could also alter the total NF-*κ*B protein content. However, derivative **2** (100 *μ*M) did not exert any effects on NF-*κ*B levels during 24-96 h incubation (Figure 6(b)). Since the role of NF-*κ*B in neurodegenerative diseases is not yet fully understood, it has been speculated that absolute inhibition of NF-*κ*B may not be even a desirable property for a neuroprotective drug [39]. For example, suppression of NF-*κ*B has been reported to increase the levels of A*β*_1-42_. Curiously, it has been shown recently that FA itself can inhibit the PI3K/Akt/NF-*κ*B signaling pathway [41]. Therefore, it was concluded that unlike FA itself, FA-derivative **2** did not have any effects on NF-*κ*B, although it was able to inhibit mTOR expression.

## 4. Discussion

This study shows that FA-derivatives **1**-**3** had many times higher uptake into the astrocytes than FA itself, especially at low concentrations, and it was primarily mediated via LAT1. Since astrocytes are known to be involved in both the inflammation and regulation of oxidative stress within the brain, they have a crucial role not only in pathophysiological changes of AD but also in other neurodegenerative diseases [20, 39, 42, 43]. Furthermore, utilizing LAT1 as a brain- and astrocyte-targeted carrier is a feasible approach to treat AD, since we have reported earlier that neither AD-induced alterations of transgenic mice nor lipopolysaccharide- (LPS-) induced inflammation changes the expression or function of LAT1 at the BBB or primary astrocytes [29, 44]. The cellular uptake was highest with derivative **3**, which was able to autoactivate the transporter, followed by derivative **2**, which in turn was able to utilize secondary transport mechanisms, namely, OATPs or OATs. Derivative **1** had the lowest transport rate, but had the highest selectivity towards LAT1. In contrast, the intracellular unbound drug concentrations of the FA-derivatives in astrocytes showed that derivative **1** had higher cellular bioavailability at lower concentrations. However, it needs to be remembered that the determination of *K*_p,uu_ values from cells assumes that the extracellular concentrations of FA-derivatives are completely unbound, and these may not correlate with the brain extracellular fluids [45]. Therefore, this result needs to be interpreted with caution. Furthermore, the studied cells were mouse primary astrocytes, and thus, the correlation to the corresponding human cells should be examined in the future to evaluate the full potential of these compounds in the human brain.

**Figure 7:**
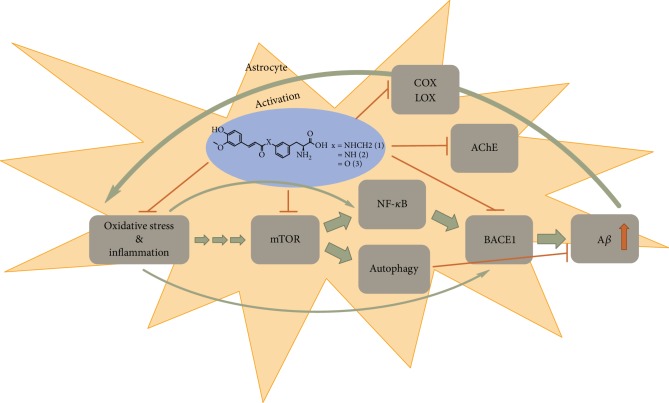
A schematic presentation of the effects of FA-derivatives **1**-**3** on biological markers that can decrease the levels of A*β* in astrocytes in AD. A*β*: *β*-amyloid; AChE: acetylcholine esterase; BACE1: *β*-site amyloid precursor protein (APP) cleaving enzyme 1; COX: cyclooxygenases; LOX: lipoxygenases; mTOR: mammalian (or mechanistic) target for rapamycin (mTOR); NF-*κ*B: transcription factor NF-*κ*B.

In addition to being potential antioxidants and preventing oxidative stress in astrocytes, FA-derivatives **1**-**3** also inhibited the function of BACE1, AChE, COX, and/or LOX, and one of the FA-derivatives also reduced the total amount of mTOR protein (Figure 7). Although AChE is mainly expressed in neurons and synapses, the levels of AChE have also been increased in primary astrocytic cultures treated with A*β*_1-42_, indicating that astrocytes may make a crucial contribution to the increased AChE activity, which can be detected in amyloid deposits in the AD brain [46]. Similarly, in addition to being present in neurons, BACE1 has also been found in activated astrocytes, which may have a crucial role in the accumulation of A*β* peptide levels and consequently on the neurodegeneration in AD [47]. On the other hand, COX enzymes are expressed in both glia cell types, i.e., astrocytes and microglia, and it has been reported that inhibition of COX-2 and 5-LOX in both cell types can significantly reduce the expression of multiple proinflammatory cytokines, such as TNF-*α* and IL-1*β* [48]. Together with decreased levels of eicosanoids, the reduced levels of cytokines have been reported to reduce the BACE1 activity and consequently A*β* production [49]. Therefore, the FA-derivatives that can accumulate into the astrocytes may have clinical relevance at multiple levels in AD: firstly by inhibiting AChE activity and increasing subsequently acetylcholine levels, secondly by inhibiting BACE1 and decreasing A*β* accumulation, and thirdly by reducing both oxidative stress and inflammation which can directly and indirectly inhibit BACE1 activation and the further production of A*β*. Together with increased autophagy mediated via mTOR inhibition, these compounds may have a significant potential to reduce A*β*-induced activation of astrocytes and, thus, block the self-sustaining cycle initiated by the accumulation of A*β* in AD (Figure 7) [39, 50]. However, it needs to be kept in mind that these efficacy results are only preliminary and obtained with a relatively high concentration against their target proteins. Therefore, more detailed experiments, such as *in vitro* compound potency studies, e.g., assessing half maximal inhibitory concentrations (IC_50_) and *in vivo* efficacy experiments with multiple doses should be carried out. In addition, the *in vitro-in vivo* (IVIV) correlation between these experiments should be examined to reveal the full potential of these derivatives towards each of the target proteins.

Although FA is a potential therapeutic agent to treat neurodegenerative diseases, such as AD, and also other neuroinflammatory disorders [51], its low BBB permeation and intense first pass metabolism limit its bioavailability and thus reduce its potential for treating human brain diseases [52, 53]. We have previously shown that converting FA to LAT1-utilizing derivatives **1**-**2** can significantly improve the BBB permeation rate, resulting in nearly 10 times greater brain exposure as measured by AUC_brain_ values and even higher, i.e., 30–120 times greater, *K*_p,brain_ values (AUC_brain_/AUC_plasma_), respectively [19]. Since FA-derivatives **1** and **2** were found to be very stable *in vivo*, they can be considered brain-targeted multifunctional therapeutic agents, whereas derivative **3** is a prodrug and is less stable, releasing FA *in vivo*. However, even though derivative **3** was bioconverted quickly in mouse plasma after i.v. administration, the *in vitro* human data showed that the hepatic intrinsic clearance was 10 times slower than that occurring in mouse, and the bioconversion in human plasma was rather insignificant. This suggests that compound **3** may have the potential to deliver, i.e., transport and release, FA to the human brain and thus act as a prodrug of FA.

Although the prodrug approach has proven to be feasible in many cases of brain targeting as exemplified by L-dopa, a successful prodrug requires not only targeted delivery but also quantitative release of the parent drug inside the target cells. Therefore, stable FA-derivatives **1** and **2**, which can elicit pharmacological effects themselves, may be more promising drug candidates for further preclinical testing as compared to FA-prodrug **3**. This study highlights a very important fact in drug design that converting a parent drug to transporter-utilizing derivative does not necessarily change the drug's activity towards its target protein. In the best case scenario, the modification may even broaden the compound's applicability and improve its potency towards other target proteins. This would be a very desirable feature, particularly in a drug intended to treat diseases like AD, in which the pathogenesis involves several distinct biochemical changes in the brain.

## 5. Conclusions

In conclusion, this study demonstrates that LAT1-utilizing derivatives of FA can improve the cellular uptake and thus they have better bioavailability in astrocytes, but they also have multifunctional therapeutic effects themselves. This can be especially beneficial in AD, but possibly also in the treatment of other neurodegenerative diseases or brain diseases in which neuroinflammation is involved. Moreover, this study proves that converting a parent drug into a transporter-utilizing derivative does not necessarily impair the potency of the compound towards its target protein; instead, the structural modification may broaden the spectrum of pharmacological effects elicited by the derivative.

## Figures and Tables

**Table 1:** Ability of ferulic acid (FA) and its derivatives **1**-**3** to bind to LAT1 in mouse primary astrocytes presented as half maximal inhibitory concentrations (IC_50_ values) of LAT1-probe substrate, [^14^C]-L-leucine uptake. Data are presented as the mean ± SD; *n* = 3-4.

Compound	IC_50_ in astrocytes (*μ*M)
FA-derivative **1**	2.15 ± 1.12
FA-derivative **2**	8.90 ± 1.25
FA-derivative **3**	28.74 ± 1.15
FA	No inhibition

**Table 2:** Michaelis-Menten kinetic parameters calculated from Eadie-Hofstee plot analysis for transporter-mediated cellular uptake of FA and its derivatives **1**-**3** into primary astrocytes. The data are presented as the mean ± SD; *n* = 3.

Compound	Primary transport mechanism (LAT1)		Primary transport mechanism (OATP/OAT)	
	*V*_max_ (nmol/min/mg protein)	*K*_m_ (*μ*M)	*V*_max_ (nmol/min/mg protein)	*K*_m_ (*μ*M)
FA-derivative **1**	0.008 ± 0.002	4.1 ± 2.1	0.033 ± 0.402	101.1 ± 19.7
FA-derivative **2**	0.013 ± 0.001	4.2 ± 0.8	0.172 ± 0.013	153.8 ± 15.0
FA-derivative **3**	Autoactivation			
FA	Autoactivation			

**Table 3:** Inhibition of AChE and BChE by ferulic acid and its derivatives **1**-**3** in mouse brain S9 subcellular fraction presented as IC_50_ values (mean ± SD, *n* = 3). Tacrine and donepezil were used as known positive controls.

Compound (*n* = 3-4)	AChE IC_50_ value (*μ*M)	BuChE IC_50_ value (*μ*M)
FA	No inhibition	No inhibition
FA-derivative **1**	16.33 ± 1.52	220.80 ± 1.14
FA-derivative **2**	25.21 ± 1.43	No inhibition
FA-derivative **3**	66.21 ± 1.23	236.10 ± 1.26
Donepezil	0.017 ± 0.001	20.74 ± 1.35
Tacrine	0.12 ± 1.10	24.12 ± 1.46

## Data Availability

The data of cellular uptake, viability and inhibition of selected biological processes used to support the findings of this study are included within the article and supplementary information file(s).

## Supplementary Materials

Supplementary Materials Supporting Information: Figure S1: uptake of ferulic acid and its LAT1-utilizing derivatives into astrocytes via LAT1. Cellular uptake of ferulic acid (A) and its LAT1-utilizing derivatives (**1**-**3**; B-C) into primary astrocytes over a concentration range 1-100 *μ*M (left) and the Eadie-Hofstee plots for transporter-mediated uptake mechanisms (right). The data is presented as the mean ± SD (*n* = 3). Table S1: intracellular unbound concentrations of ferulic acid and its LAT1-utilizing derivatives. Unbound fraction (*f*_u,cell_) (%) of FA and its LAT1-utilizing derivatives **1**-**3**. The data is presented as the mean ± SD (*n* = 3). Figure S2: ability of ferulic acid and its LAT1-utilizing derivatives to inhibit astrocyte cell growth. The remaining cell viability of primary astrocytes after 72 h incubation of 1-400 *μ*M ferulic acid (A) and its LAT1-utilizing derivatives **1** (B), **2** (C), and **3** (D) presented as percentages (%) compared to the untreated cells (ctrl) (mean ± SD, *n* = 3‐6). An asterisk denotes a statistically significant difference from the respective control (^∗∗∗^*P* < 0.001, one-way ANOVA, followed by Tukey's test). Figure S3: ability of ferulic acid and its LAT1-utilizing derivatives to inhibit AChE/BuChE activity. Hanes-Woolf's plots of AChE activity in the presence of 16-66 *μ*M FA-derivatives **1** and **3** (A-B, respectively; open circles) compared to the control (filled circles).
