# Supplementary material for: Astrocyte-Targeted Transporter-Utilizing Derivatives of Ferulic Acid Can Have Multifunctional Effects Ameliorating Inflammation and Oxidative Stress in the Brain
Source: Oxid Med Cell Longev. 2019 Nov 13;2019:3528148. doi: 10.1155/2019/3528148 (PMC6877910; doi:10.1155/2019/3528148)
Supplement: Supplementary Materials — Supporting Information: Figure S1: uptake of ferulic acid and its LAT1-utilizing derivatives into astrocytes via LAT1. Cellular uptake of ferulic acid (A) and its LAT1-utilizing derivatives (1-3; B-C) into primary astrocytes over a concentration range 1-100 μM (left) and the Eadie-Hofstee plots for transporter-mediated uptake mechanisms (right). The data is presented as the mean ± SD (n = 3). Table S1: intracellular unbound concentrations of ferulic acid and its LAT1-utilizing derivatives. Unbound fraction (fu,cell) (%) of FA and its LAT1-utilizing derivatives 1-3. The data is presented as the mean ± SD (n = 3). Figure S2: ability of ferulic acid and its LAT1-utilizing derivatives to inhibit astrocyte cell growth. The remaining cell viability of primary astrocytes after 72 h incubation of 1-400 μM ferulic acid (A) and its LAT1-utilizing derivatives 1 (B), 2 (C), and 3 (D) presented as percentages (%) compared to the untreated cells (ctrl) (mean ± SD, n = 3‐6). An asterisk denotes a statistically significant difference from the respective control (∗∗∗P < 0.001, one-way ANOVA, followed by Tukey's test). Figure S3: ability of ferulic acid and its LAT1-utilizing derivatives to inhibit AChE/BuChE activity. Hanes-Woolf's plots of AChE activity in the presence of 16-66 μM FA-derivatives 1 and 3 (A-B, respectively; open circles) compared to the control (filled circles). [file 3528148.f1.pdf]

Supporting Information

Astrocyte-Targeted Transporter-Utilizing Derivatives of Ferulic Acid Can Have Multifunctional Effects Ameliorating Neuroinflammation and Oxidative Stress in the Brain

Ahmed Montaser,[1,#] Johanna Huttunen,[1,#] Sherihan Abdelhamid Ibrahim,[2] Kristiina M. Huttunen [1,]*

[1] School of Pharmacy, Faculty of Health Sciences, University of Eastern Finland,

P.O. Box 1627, FI-70211 Kuopio, Finland

[2] Department of Pharmacology and Therapeutics, Faculty of Pharmacy and Drug Manufacturing,

Pharos University, 21311 Alexandria, Egypt

* Corresponding author, E-mail: kristiina.huttunen@uef.fi

Contents

1. *Uptake of Ferulic Acid and Its LAT1-Utilizing Derivatives into Astrocytes via LAT1* 2

2. *Intracellular Unbound Concentrations of Ferulic Acid and Its LAT1-Utilizing Derivatives* 3

3. *Ability of Ferulic Acid and Its LAT1-Utilizing Derivatives to Inhibit Astrocyte Cell Growth* 4

4. *Ability of Ferulic Acid and Its LAT1-Utilizing Derivatives to Inhibit AChE/BuChE Activity* 5

1. Uptake of Ferulic Acid and Its LAT1-Utilizing Derivatives into Astrocytes via LAT1

A) Ferulic Acid (FA)

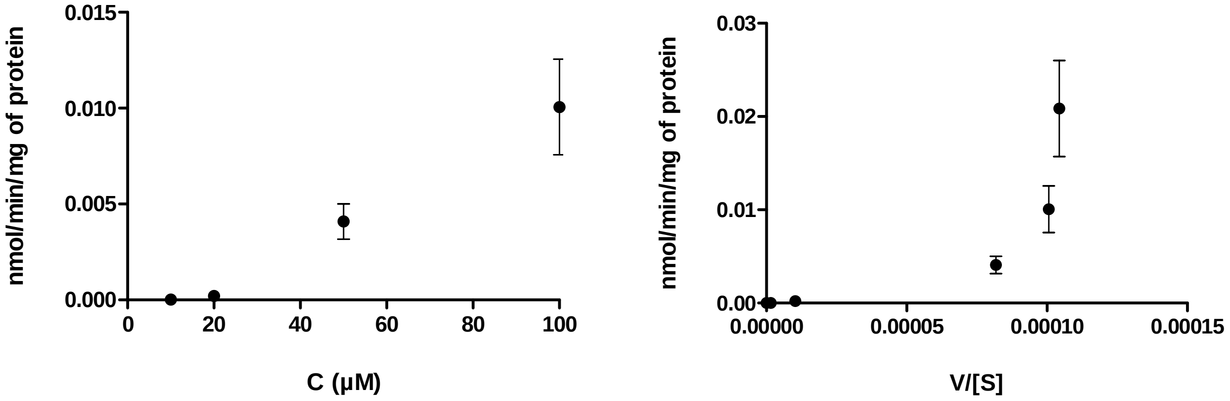

B) Derivative 1

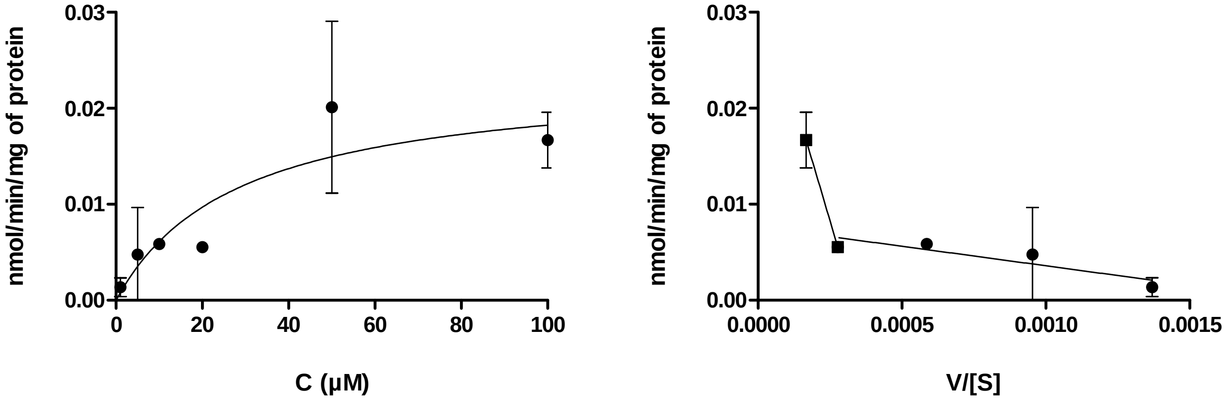

C) Derivative 2

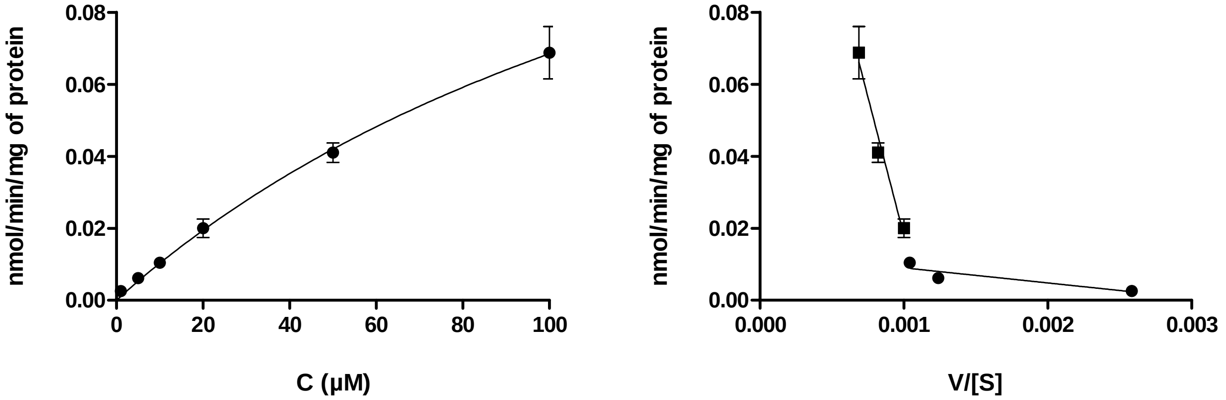

D) Derivative 3

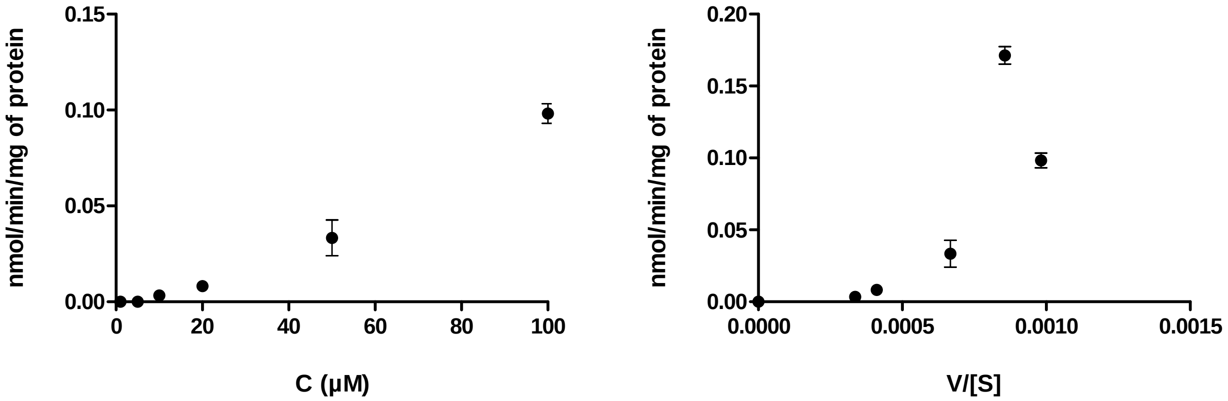

Figure S1. Cellular uptake of ferulic acid (**A**) and its LAT1-utilizing derivatives (**1-3; B-C**) into primary astrocytes over a concentration range 1-100 µM (left) and the Eadie-Hofstee plots for transporter-mediated uptake mechanisms (right). The data is presented as mean ± SD (n=3).

2. *Intracellular Unbound Concentrations of Ferulic Acid and Its LAT1-Utilizing Derivatives*

Table S1. Unbound Fraction, $f_{u,cell}$ (%) of FA And Its LAT1-Utilizing Derivatives **1-3**. The data is presented as mean ± SD (n=3).

**Compound**	**$f_{u,cell}$ (%)**
**FA**	*Fully unbound*
**FA-Derivative 1**	16.26 ± 2.72
**FA-Derivative 2**	8.06 ± 0.54
**FA-Derivative 3**	*Cannot be evaluated due to the metabolism*

3. Ability of Ferulic Acid and Its LAT1-Utilizing Derivatives to Inhibit Astrocyte Cell Growth

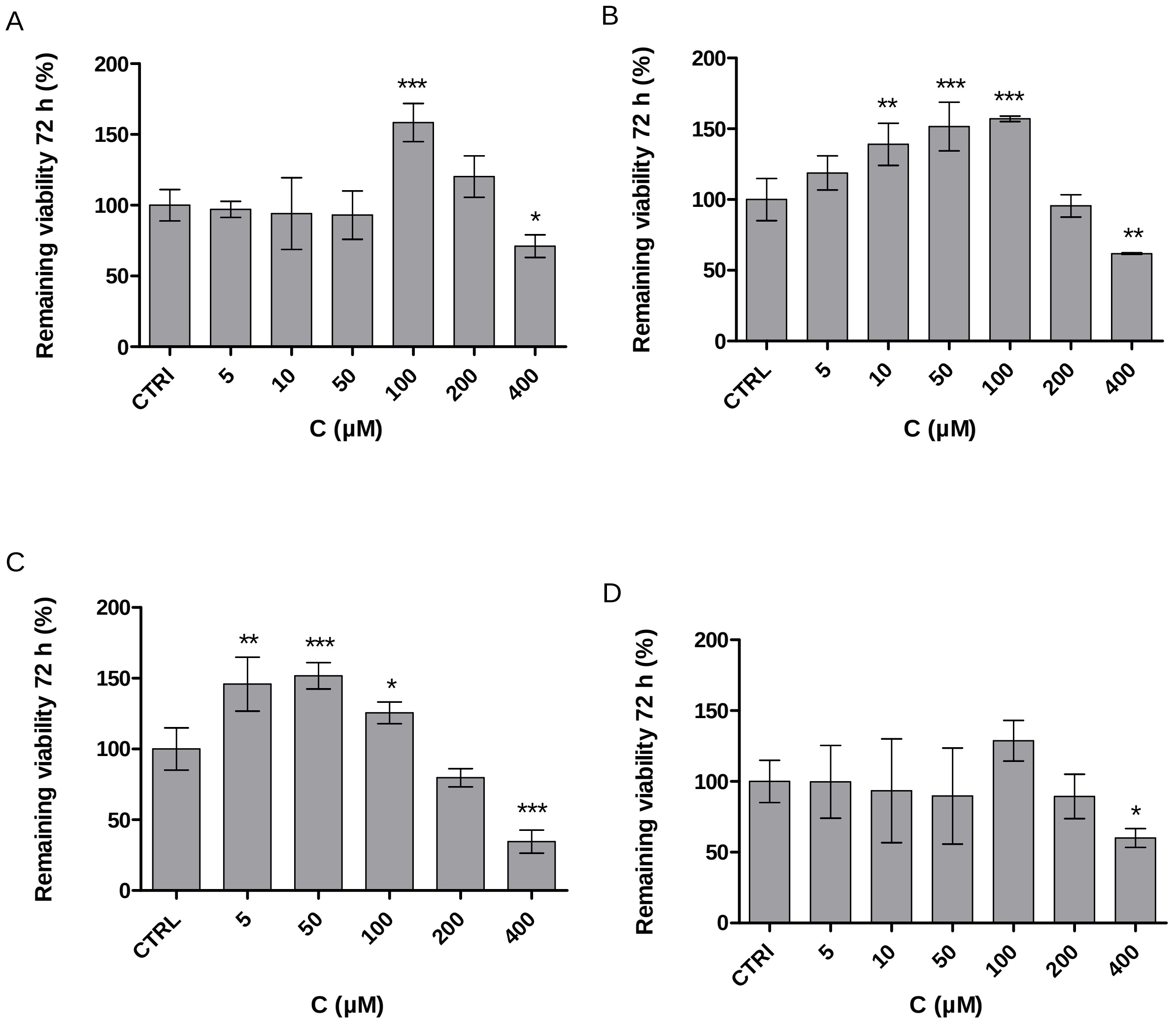

Figure S2. The remaining cell viability of primary astrocytes after 72 h incubation of 1-400 µM ferulic acid (**A**) and its LAT1-utilizing derivatives **1** (**B**), **2** (**C**) and **3** (**D**) presented as percentages (%) compared to the untreated cells (ctrl) (mean ± SD, n=3-6). An asterisk denotes a statistically significant difference from the respective control (*** $P < 0.001$, one-way ANOVA, followed by Tukey's test).

4. *Ability of Ferulic Acid and Its LAT1-Utilizing Derivatives to Inhibit AChE/BuChE Activity*

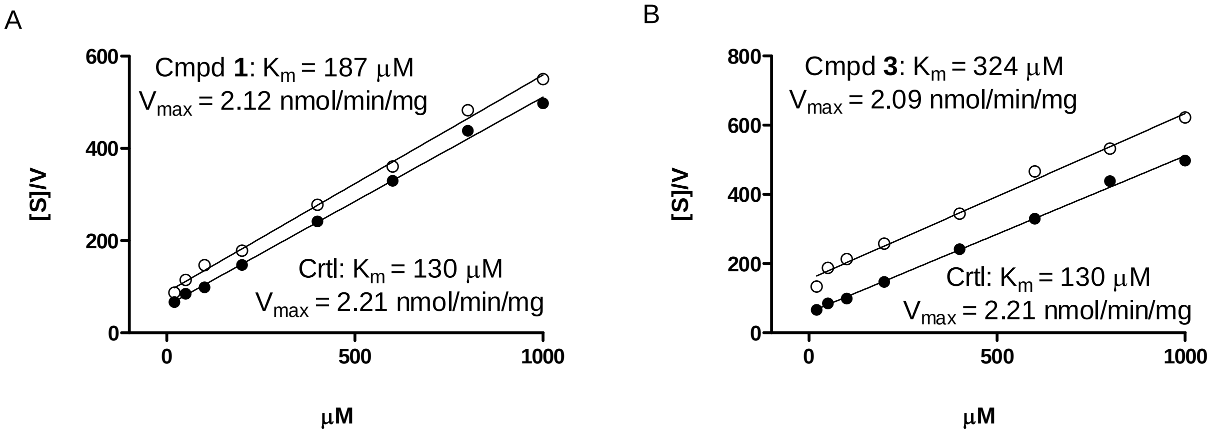

Figure S3. Hanes-Woolf's plots of AChE activity in the presense of 16-66 μM FA-derivatives **1** and **3** (**A**-**B**, respectively; open circles) compared to the control (filled circles).
